# Protocol to study metastatic dormancy using 4T07-mCherry breast cancer cells and an algorithm-based quantification of disseminated cancer cells

**DOI:** 10.1016/j.xpro.2025.104182

**Published:** 2025-11-03

**Authors:** Paulo Pereira, Virginia Cecconi, Maries van den Broek

**Affiliations:** 1 Institute of Experimental Immunology, University of Zurich, Zurich, Switzerland

**Keywords:** Cancer, Immunology, Microscopy

## Abstract

Dormant disseminated cancer cells are responsible for late relapses of breast cancer. Here, we present a protocol to orthotopically inject 4T07-mCherry breast cancer cells into mice and describe how to resect the primary tumor. We detail steps for collecting and processing the lungs to detect and quantify cancer cells using an algorithm-based approach. This protocol allows studying the induction, maintenance, and awakening from metastatic dormancy.

For complete details on the use and execution of this protocol, please refer to Pereira et al.^1^

## Highlights

• Steps for injecting breast cancer cells in the mammary fat pad without surgery
• Instructions to surgically resect a breast primary tumor
• Guidance on collecting and processing lungs for immunofluorescence analysis
• Steps to quantify disseminated cancer cells in the lungs with an algorithm-based approach

## Abstract (editor highlights)

Publisher’s note: Undertaking any experimental protocol requires adherence to local institutional guidelines for laboratory safety and ethics.

## Graphical abstract

**Figure:**
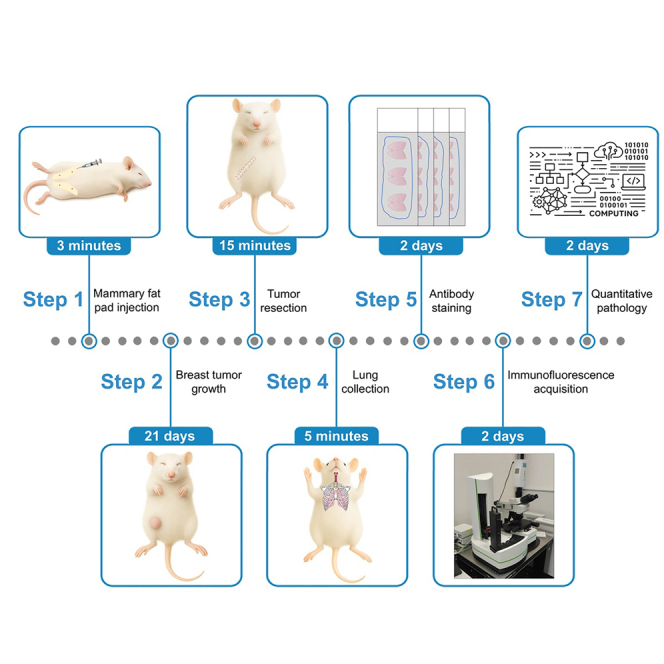

## Before you begin

Metastases originate from disseminated cancer cells (DCCs) that detach from the primary tumor and seed distant organs. These DCCs can enter a long-term dormant state, known as metastatic dormancy.

We developed a model of metastatic dormancy in immunocompetent BALB/c mice using mCherry-labeled 4T07 breast cancer cells (4T07-mCherry). In this system, cancer cells spread from the primary tumor to the lungs, where they persist in a quiescent state.^2^^,^^3^ We identified and quantified these DCCs through an algorithm-based quantitative protocol, which can be adapted to detect and quantify other structures and cells of interest.^4^

This model offers two key advantages: First, the use of immunocompetent mice enables investigating, how the innate and adaptive immune systems regulate metastatic dormancy. Second, fluorescent labeling allows for sensitive detection and quantification of DCCs through algorithm-based quantitative pathology.

### Innovation

This protocol combines distinct techniques into a single workflow that allows users to study the sequential steps of metastatic dormancy, namely the induction, maintenance, and awakening.

We provide a detailed description of the culture, preparation and injection of cancer cells, the resection of the primary tumor, and the collection of lungs in mice. Further, we provide a comprehensive protocol for processing and staining the lungs with fluorophore-labeled antibodies. Finally, we provide the technical details to analyze the immunofluorescence data and identify/quantify cells of interest.

The techniques described in this protocol can also be used separately for experiments outside the scope of metastatic dormancy. Altogether, our protocol provides the user with the experimental details to correctly perform mouse experiments to address metastatic dormancy and the technical instructions to analyze the generated data.

### Institutional permissions

All experiments were performed according to the Swiss cantonal and federal regulations on animal protection and approved by the Cantonal Veterinary Office Zurich under the license ZH026/2021.

### Preparation of 4T07-mCherry breast cancer cells for injection

**Timing: 30 min**

1. Collecting expanded 4T07-mCherry cells *in vitro.*
  a. Confirm cells are 60%–90% confluent.
    ***Note:*** 4T07-mCherry cells are cultured in complete DMEM (DMEM supplemented with 5% fetal bovine serum, 2 mM L-glutamine, 100 U/mL penicillin and 100 μg/mL streptomycin). 4T07-mCherry cells are freshly thawed and cultured in a T75 flask before each experiment. Cells are split once after thawing before injection. To increase reproducibility, cells are thawed from a stock of many replica vials.
    **CRITICAL:** The cells are tested negative for *Mycoplasma* and other mouse pathogens.
  b. Aspirate the medium.
  c. Rinse the adherent cells carefully with 5 mL of PBS.
    ***Note:*** Do not add PBS directly onto the cells. This may lead to cell detachment and consequent loss of cells upon washing.
  d. Close the flask and place it horizontally on the bench.
  e. Gently swirl the flask.
  f. Aspirate the PBS.
  g. Add 3 mL of trypsin.
  h. Close the flask and place it horizontally on the bench.
  i. Gently swirl the flask.
  j. Incubate at 37°C for 5 minutes.
  k. Collect the detached cells by gently pipetting up and down with complete DMEM.
  l. Centrifuge at *350 g* for 5 minutes at 4°C.
  m. Aspirate the supernatant.
2. Preparing 4T07-mCherry cells for *in vivo* injection.
  a. Resuspend the pellet in 1 mL of ice-cold PBS.
  b. Take an aliquot of cells and mix it 1:1 with trypan blue (0.4% in PBS).
    ***Note:*** Dead cells appear blue.
  c. Count the cells using a hemocytometer (or an automated cell counter).
    ***Note:*** Approximately 4x10^6^ live 4T07-mCherry cells are expected per T75 flask.
  d. Adjust the density to 2.0x10^6^ live cells/mL with ice-cold PBS (injection mix).
    ***Note:*** Each mouse will be injected with 50 μL of the injection mix (10^5^ 4T07-mCherry cells). Prepare 20% more volume of the injection mix than needed (maintaining the final concentration of 2.0x10^6^ live cells/mL) as a safety net. Use round-bottom tubes to prevent cell sedimentation.
  e. Keep the cells on ice and immediately proceed to the injection.

### Preparing the mouse for the injection of 4T07-mCherry cells

**Timing: 5 min per mouse**

**Figure 1:**
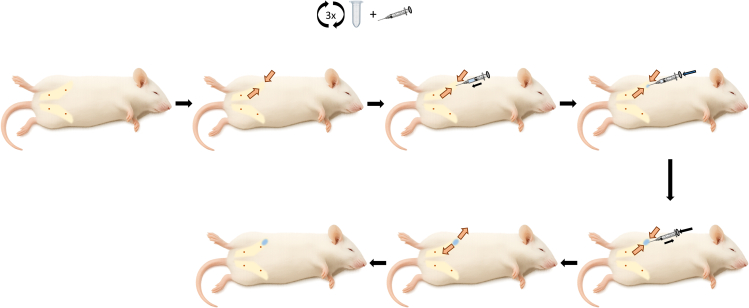
Injection of 4T07-mCherry cells into the 4^th^ right mammary fat pad of a female BALB/c mouse Experimental procedure to inject cancer cells in the mammary fat pad. Expose the 4^th^ mammary fat pad by applying pressure with the thumb and index finger on the nipple and the skin opposite to it, respectively. Horizontally insert an insulin syringe with 50 μL of PBS containing 10^5^ cancer cells and slowly inject into the 4^th^ mammary fat pad. Remove the syringe while maintaining the pressure on the plunger. Release the pressure applied with the thumb and index finger.

3. Positioning the mouse for the identification of the 4^th^ right mammary fat pad.
  a. Transfer a female BALB/c mouse into an induction box with 3% isoflurane and an oxygen flow rate of 0.8 Liter/minute.
  b. Wait until the mouse is under anesthesia.
    ***Note:*** This takes 1-3 minutes, depending on the size of the induction box.
  c. Gently invert the tube containing the injection mix 3 times.
  d. Load 50 μL of the injection mix (containing 10^5^ 4T07-mCherry cells) into an insulin syringe (30G, 12 mm needle).
  e. Remove the mouse from the induction box.
  f. Place the mouse onto its left flank on a sterile surface, with its head towards the right side of the table (Figure 1).

### Preparation of the mouse for surgery

**Timing: 5 min per mouse**

**Figure 2:**
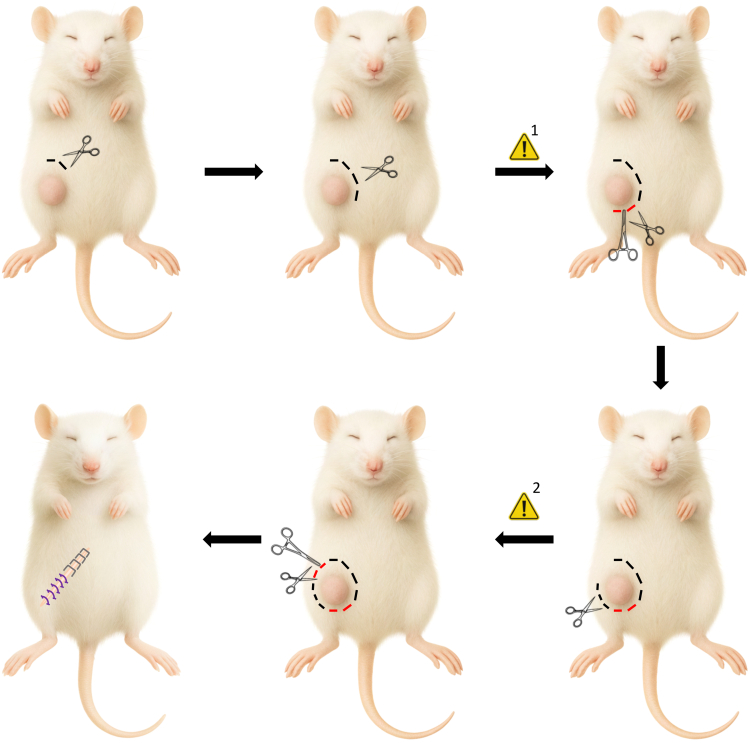
Resection of the primary breast tumor in the 4^th^ right mammary fat pad Experimental procedure to remove a primary breast tumor from the 4^th^ right mammary fat pad. Starting from the cranial end of the tumor, cut the skin clockwise until the right caudal end of the tumor. Clamp and cut above the blood vessel connected to the tumor. Continue cutting clockwise until the left cranial end of the tumor. Clamp and cut below the blood vessel connected to the tumor and remove the tumor-draining lymph node. Excise the tumor and staple the cranial region of the wound (near the chest) and stitch the caudal region of the wound (near the leg). ^1^ and ^2^ signal for the presence of a blood vessel near the tumor, marked by red dashes. Staples are represented in gray and stitches in purple.

4. Preparing the mouse for surgery.
  a. Transfer one mouse into an induction box with 3% isoflurane and an oxygen flow rate of 0.8 Liter/minute.
    ***Note:*** Ensure the mouse is anesthetized before proceeding to the next step by confirming the absence of the pedal reflex.
    **CRITICAL:** All the following steps must be performed on an operating bench/warm pad at 37°C to prevent hypothermia and death.
  b. Subcutaneously inject 0.04 mg/kg of fentanyl into the neck using an insulin syringe (30G, 8 mm needle).
  c. Place the mouse on its back onto the bench and insert its snout in a tube connected to 3% isoflurane and an oxygen flow rate of 0.8 Liter/minute.
  d. Apply ophthalmic ointment (Alcon) to the eyes to prevent them from drying.
  e. Shave the lower right quadrant of the abdomen using hair clippers (Isis, Aesculap).
    ***Note:*** Shaving is sufficient to expose the skin; the use of depilation cream is unnecessary.
  f. Disinfect the shaved area with a tissue dampened with 70% ethanol.
  g. Confirm the absence of the pedal reflex once more.
  h. Perform the surgery (Figure 2).

### Preparation of the lungs for freezing

**Timing: 3 days**

**Figure 3:**
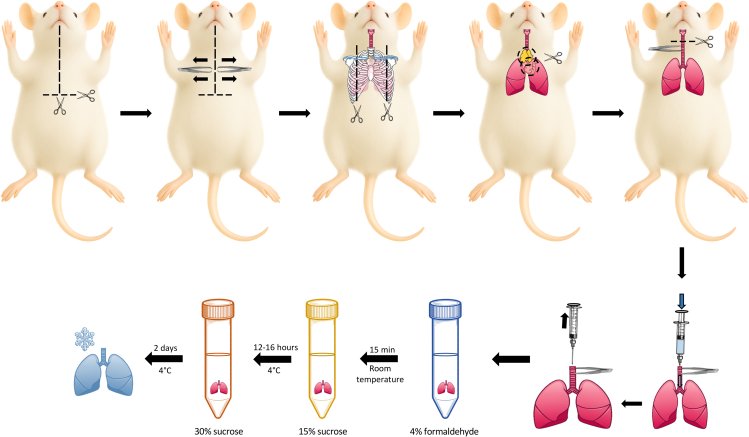
Collection and inflation of the lungs Overview of the steps to collect the lungs. Remove the skin on the thorax and expose the rib cage. Cut the diaphragm and the bones of the rib cage to expose thymus, heart and lungs. Remove the rib cage, the thymus and the heart. Transversely cut the trachea and remove the lungs from the thorax. Inflate the lungs with 4% formaldehyde and perform sequential incubations on 4% formaldehyde, 15% sucrose, and 30% sucrose. Freeze the lungs in O.C.T. Compound.
  e. Still holding the trachea, transfer the lungs to a 50-mL tube pre-filled with 4% formaldehyde in PBS at 20°C–25°C (Figure 3).
  f. Incubate for 15 minutes at 20°C–25°C.
  g. Transfer the lungs into a 50-mL tube containing 15% sucrose in PBS at 4°C.
  h. Incubate 12–16 hours at 4°C.
  i. Transfer the lungs into a 50-mL tube containing 30% sucrose in PBS at 4°C.
  j. Incubate for 2 days at 4°C.

5. Fixing and dehydrating the lungs.
  a. Fill a 5-mL syringe with 4% formaldehyde in PBS and connect it to an intravenous plastic cannula.
  b. Insert the plastic cannula into the trachea (Figure 3).
  c. Adjust the hold with the tweezers so that the tweezers press the trachea against the plastic gauge.
    **CRITICAL:** This step is crucial to prevent the backflush of formaldehyde upon inflation. Adjust the pressure on the tweezers to the malleability of the cannula to allow the flow of formaldehyde into the trachea.
  d. Inflate the lungs with 2-3 mL of 4% formaldehyde in PBS (Figure 3).
    ***Note:*** Both lungs increase in size during inflation. For problems with inflating the lungs, see Troubleshooting: Problem 3.

### Freezing the lungs

**Timing: 30 min**

6. Freezing the lungs in O.C.T. compound.
  a. Pour O.C.T. Compound (Sakura) into the lid of a 50-mL tube.
    ***Note:*** Use sufficient O.C.T. Compound to completely cover the lungs.
  b. Transfer the lungs from the 30% sucrose into the lid with O.C.T. Compound.
  c. Cut off the trachea from the lungs.
  d. Fill ¾ of a Tissue-Tek Standard Cryomold (Sakura) with O.C.T. Compound.
  e. Transfer the lungs into the cryomold with O.C.T. Compound.
    ***Note:*** Place the lungs in the cryomold so that the ventral part is facing upwards. Avoid the formation of bubbles.
  f. Pour 100% ethanol into a Styrofoam box.
    ***Note:*** At this stage, add only enough ethanol to evenly cover the bottom of the box.
  g. Add dry ice pellets to the ethanol, spacing them regularly over the bottom of the box.
  h. Pour more 100% ethanol into the Styrofoam box.
    ***Note:*** Adjust the amount of ethanol so that the cryomolds will sit on the base of the Styrofoam box but not float.
  i. Wait 1 minute for the 100% ethanol to cool down.
  j. Pour more O.C.T. Compound onto the lungs to completely cover them.
    ***Note:*** Gently press the lungs down to completely submerge them. Avoid the formation of bubbles.
  k. Place the cryomold containing the lungs onto the bottom of the box.
  l. Wait for the O.C.T. Compound to completely freeze.
  m. Remove the cryomold with the frozen lungs.
  n. Pat the cryomold dry with a tissue to remove the ethanol.
  o. Store the lungs at −80°C until cutting.
    ***Note:*** Ideally, leave the frozen lungs for at least a couple of hours at −80°C before proceeding to the cryotome to ensure the whole block is at the same temperature.

### Preparation for staining the lung sections with antibodies

**Timing: 30 min**

7. Preparing the solutions and antibody mixes.
  a. Prepare a batch of 4% BSA in PBS and 1% BSA in PBS.
    ***Note:*** Aliquot these solutions into separate 2-mL tubes and store at −20°C until use.
  b. Prepare the permeabilization solution, consisting of 4% BSA and 0.01% Triton X-100 in PBS.
    ***Note:*** Aliquot this solution into 2-mL tubes and store at −20°C until use.
  c. Prepare the washing buffer, consisting of 0.05% Tween 20 in PBS.
    ***Note:*** Prepare 10 L of this buffer and store at 20°C–25°C.
  d. Prepare the primary antibody mix in PBS containing 1% BSA, as follows:
    i. Unlabeled goat anti-mCherry antibody, 1:400.
    ii. Unlabeled rabbit anti-Ki67 antibody, 1:100.
    iii. Labeled rat anti-CD45 in CoraLitePlus 647, 1:400.
      ***Note:*** Always prepare this solution on the day of staining.
  e. Prepare the secondary antibody mix in PBS containing 1% BSA, as follows:
    i. Donkey anti-goat antibody, 1:200.
    ii. Donkey anti-rabbit antibody, 1:200.
  f. Prepare the nucleus staining solution by diluting DAPI in water (1:5000, Thermo Fisher).

### Setting up a protocol for the detection of immunofluorescence

**Timing: 1 h**

**Figure 4:**
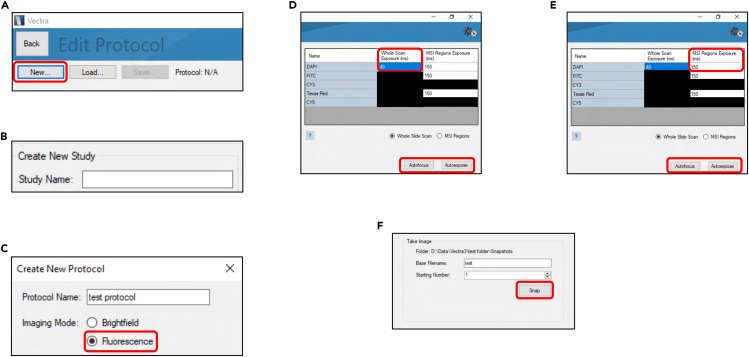
Setting up the protocol for the detection of immunofluorescence Sequential steps to create a protocol with optimized exposure times for different fluorophores. Images adapted from screenshots of the software *Vectra 3.0.7.*
  c. Create a new study, which corresponds to the folder where the protocol will be saved (Figure 4B).
  d. Name your protocol and select “Imaging Mode: Fluorescence” (Figure 4C).
  e. Save the protocol in the study created in step 9c.

8. Acquiring the fluorescence references.
  a. Open the software *Vectra 3.0.7.*
  b. Select “Check Dashboard”.
  c. Select “Fluorescence References”.
  d. Acquire the fluorescence references with the slide provided for that effect.
9. Creating a protocol and a study (folder).
  a. Go back to the initial dashboard of *Vectra 3.0.7.*
  b. Select “Edit Protocol” and “New…” (Figure 4A).

## Key resources table

**Table:** 

REAGENT or RESOURCE	SOURCE	IDENTIFIER
**Antibodies**		

Donkey anti-goat in Alexa Fluor 594 (polyclonal), 1:200	Jackson ImmunoResearch	Cat# 705-585-147, RRID:AB_2340433
Donkey anti-rabbit in Alexa Fluor 488 (polyclonal), 1:200	Jackson ImmunoResearch	Cat# 711-545-152, RRID:AB_2313584
Goat anti-mCherry (polyclonal), 1:400	SICGEN	Cat# AB0040, RRID:AB_2333093
Rabbit anti-Ki67 (monoclonal), 1:100	Abcam	Cat# ab16667, RRID:AB_302459
Rat anti-CD45 in CoraLite Plus 647 (monoclonal), 1:400	Proteintech	Cat# CL647-65087, RRID:AB_2883673

**Chemicals, peptides, and recombinant proteins**		

4% formaldehyde	Carl Roth	P087.4
Buprenorphine	Schering-Plough	N/A
Fentanyl	Kantonsapotheke Zurich	N/A
Tissue-Tek O.C.T. compound	Sakura	4583
ProLong Diamond antifade mountant	Invitrogen	P36961
Tween 20	Sigma-Aldrich	P1379

**Experimental models: Cell lines**		

Female 4T07-mCherry	Laboratory of Maries van den Broek^1^	N/A

**Experimental models: Organisms/strains**		

Female BALB/cJRj mouse (8–10 weeks old at the time of the injection of cancer cells)	Janvier Labs	N/A

**Software and algorithms**		

inForm 2.6.0	Akoya Biosciences	https://www.akoyabio.com/phenoimager/inform-tissue-finder/
Phenochart 1.1.1	Akoya Biosciences	https://www.akoyabio.com/software-data-analysis/
Vectra 3.0.7	Akoya Biosciences	https://www.akoyabio.com/

**Other**		

5-0 coated Vicryl stitches	BD Biosciences	V303H
Autoclip wound clips	Ethicon	427631
Cordless hair clipper ISIS	Aesculap	GT420
Cryostar NX70 Cryostat	Epredia	15380755
Dulbecco’s modified Eagle’s medium (DMEM)	Gibco	11965092
Fetal bovine serum	Gibco	A5256701
ImmEdge hydrophobic barrier pen	Vector Laboratories	H-4000
Insulin syringe 0.3 mL 8 mm needle	BD Micro-Fine	324826
Insulin syringe 0.5 mL 12 mm needle	Braun	9151125S
Lacryvisc ophthalmic ointment	Alcon	1857926
Tissue-Tek Cryomold standard square (25 × 20 × 5 mm)	Sakura	25608-916

## Step-by-step method details

### Identification of the 4^th^ right mammary fat pad and orthotopic injection of 4T07-mCherry cells

**Timing: 3 min**

This section of the protocol explains how to identify and expose the 4^th^ mammary fat pad of anesthetized female BALB/c mice to orthotopically inject 4T07-mCherry cells.

1. Exposing the mammary fat pad.
  a. Place your thumb over the 4^th^ mammary fat pad nipple and the index finger on the opposite side.
  b. Gently press your fingers to expose the mammary fat pad (both fingers are represented by orange arrows in Figure 1).

**CRITICAL:** Do not squeeze the skin tightly to avoid damage.

***Note:*** Observe the exposure of a soft, spongy lump of skin between your fingers (corresponding to the mammary fat pad). For issues identifying the mammary fat pad, see Troubleshooting: Problem 1.

2. Injecting 4T07-mCherry cells into the exposed mammary fat pad.
  a. Still holding the mammary fat pad between the fingers, horizontally insert the syringe in the direction head-to-tail (Figure 1).
  b. Push the needle horizontally until its tip is close to the middle of the mammary fat pad.
    ***Note:*** Insert approximately 5 mm of the needle. Do not push the needle deeper, as this can lead to injecting outside of the 4^th^ mammary fat pad.
  c. Slowly inject 50 μL of injection mix into the 4^th^ mammary fat pad (Figure 1).
    ***Note:*** A small blob inside the mammary fat pad is often seen after the injection.
  d. Slowly remove the needle from the mammary fat pad, maintaining pressure on the plunger (Figure 1).
    ***Note:*** Maintaining the pressure on the plunger decreases the chances of backflush. A small drop can still form after removing the needle.
  e. Place the mouse back into the cage and observe until it recovers from anesthesia.
  f. Wait 21 days for the primary tumor to grow.

### Resection of the tumor and closing the wound

**Timing: 15 min**

This section describes the list of steps for the resection of the primary tumor in the 4^th^ right mammary fat pad of an anesthetized mouse and details the steps to close the resulting the wound.

3. Tumor resection.
  a. Make a small incision in the skin above the tumor (Figure 2).
    **CRITICAL:** Do not cut the peritoneum. See Troubleshooting: Problem 2.
  b. Insert the scissors under the cut skin.
  c. Cut the skin clockwise around the tumor until the blood vessel at the right caudal end of the tumor (Figure 2).
    **CRITICAL:** Do not to cut yet the blood vessel supplying the caudal end of the tumor (red dashes in Figure 2).
    ***Note:*** Leave a gap of approximately 5 mm between the cut and the primary tumor to ensure no residual primary cancer cells remain in the mouse.
  d. Clamp the blood vessel connected to the tumor with straight hemostatic forceps and cut above the forceps (closer to the tumor than to the peritoneum).
    ***Note:*** Occasionally, bleeding may occur. Gently dry the wound before proceeding. Small tumors may not have a blood vessel in this location.
  e. Continue cutting the skin around the tumor until the blood vessel at the left cranial end of the tumor (Figure 2).
    **CRITICAL:** Do not cut yet the blood vessel supplying the cranial end of the tumor (red dashes in Figure 2).
    **CRITICAL:** Near the leg, leave a gap of less than 5 mm between the cut and the primary tumor to ensure there is enough skin left on the leg for stitching.
  f. Clamp the blood vessel connected to the tumor with straight hemostatic forceps and cut under the forceps (closer to the tumor than to the peritoneum).
    ***Note:*** Occasionally, bleeding may occur. Gently dry the wound before proceeding. Small tumors may not have a visible blood vessel in this location.
    **CRITICAL:** Remove the tumor-draining lymph node next to the blood vessel.
  g. Excise the tumor.
4. Wound closure.
  a. Hold the abdominal skin on one side of the wound with a pair of curved serrated tweezers on one hand.
  b. Gently hold the peritoneum of the same side of the wound with another pair of curved serrated tweezers on the other hand.
    ***Note:*** Using serrated tweezers improves the grip.
    **CRITICAL:** Do not tightly squeeze the tweezers to prevent tissue damage.
  c. Gently pull the tweezers in opposite directions.
    ***Note:*** Pull only enough to separate the skin around the wound from the peritoneum.
  d. Repeat the process for the other side of the wound.
  e. Hold together both sides of the detached abdominal skin at the beginning of the wound (closer to the head than to the tail).
  f. Staple that junction of skin with one Autoclip wound clip (9 mm, Ethicon).
    **CRITICAL:** Never staple the skin attached to the peritoneum or the peritoneum itself.
  g. Repeat the process in the cranial-to-caudal direction until the leg-lower abdomen junction (3-4 staples in total).
    **CRITICAL:** Ensure that there is enough unstapled skin left between the lower abdomen and the leg to allow mobility.
  h. Hold the skin of the leg on one side of the wound with a pair of curved serrated tweezers on one hand.
  i. Place a curved serrated tweezer under the skin.
  j. Gently detach the skin from the muscle by slowly opening and closing the tweezers under the skin.
  k. Repeat this process for the other side of the wound.
  l. Hold together both sides of the detached skin of the leg below the last staple.
  m. Using hemostatic forceps and tweezers, stitch together both sides of the skin of the leg.
    **CRITICAL:** Never stitch the skin attached to the muscle or the muscle itself.
  n. Repeat the process until the wound is completely closed (Figure 2).
  o. Disinfect the closed wound.
  p. Subcutaneously inject 0.1 mg/kg of buprenorphine into the neck using an insulin syringe (30G, 8 mm needle).
  q. Remove the mouse from the isoflurane mask.
  r. Keep the mouse on the operating bench/warm pad until it begins to wake up.
  s. Gently place the mouse back into the cage.
    ***Note:*** Only keep mice that underwent surgery on the same day in the same cage. Do not pool them with mice that did not undergo surgery. Perform daily checks for 7 days, after which the normal health check periodicity is resumed.
    **CRITICAL:** Provide the mice with *ad libitum* access to drinking water containing 10 μg/mL of buprenorphine for 48 hours.

### Collecting the lungs from the thoracic cage

**Timing: 2 min**

This section details each step required for the safe removal of the lungs from the thoracic cage.

5. Lung collection from the thoracic cage.
  a. Cut the skin from the middle of the lower end of the thoracic cage up to the middle of the lower mandible (Figure 3).
  b. Cut the peritoneum to expose the diaphragm.
  c. Cut the diaphragm perpendicularly to the sternum.
  d. Hold the cut skin with one tweezer on each side.
  e. Pull the skin in opposite directions to expose the rib cage.
  f. Cut the outer part of the rib cage on the right side, all the way up toward and including the clavicle (marked blue) (Figure 3).
  g. Cut the outer part of the rib cage on the left side, all the way up toward and including the clavicle (marked blue) (Figure 3).
    ***Note:*** The clavicle has to be cut on both sides to allow the posterior removal of the lungs while holding the trachea.
  h. Cut the tissues and muscles anchoring both sides of the outer part of the rib cage to the body.
  i. Remove both sides of the outer part of the rib cage by pulling with tweezers.
    **CRITICAL:** Do not damage the trachea or the lungs when removing the thoracic cage, as this precludes the execution of the subsequent inflation steps.
  j. Remove the thymus and the heart (Figure 3).
    **CRITICAL:** Do not damage the trachea or lungs.
  k. Perform a transversal cut on the trachea on the upper half of the neck (Figure 3).
  l. Carefully cut the tissue underneath the trachea and lungs that connects them to the mouse.
  m. Hold the trachea with a pair of tweezers.
  n. Gently pull the trachea and lungs from the mouse.
    **CRITICAL:** Also extract the lungs from a healthy, tumor-free mouse. These lungs will be processed in parallel to the cancer cell-containing lungs in all subsequent steps.

### Preparing lung sections from frozen lungs

**Timing: 10 min**

These steps describe the workflow to section frozen lungs blocks for posterior staining with antibodies.

6. Cryosectioning frozen lung blocks.
  a. Place the frozen lung blocks in the cryotome at −20°C.
    **CRITICAL:** Allow the blocks to reach −20°C before proceeding to prevent the cut sections from curling or sticking to the blade.
  b. Fix one block to a round holder using O.C.T. Compound.
    **CRITICAL:** Ensure the O.C.T. Compound is completely frozen and well attached to the block before proceeding.
  c. Place the block on the cutting stage holder and firmly lock it.
    ***Note:*** Place the ventral part of the lungs facing the blade.
  d. Trim and discard 50 μm-thick sections of the O.C.T. Compound covering the lungs until the lungs become visible.
  e. Change the cutting thickness to 10 μm.
  f. Cut 10-μm thick sections of the lungs with a cryotome (Epredia).
    ***Note:*** Place 3 sections per slide. One section should contain the ventral part of the lungs, one section should contain the middle part of the lungs, and one section should contain the dorsal part of the lungs. This ensures that the quantification of cells in the lungs is representative of the whole lung.
  g. Incubate the slides at 37°C for 1 hour to dry.
    ***Note:*** The slides can also be dried by placing them for 12-16 hours at 20°C–25°C, protected from light.

### Staining lung sections with antibodies

**Timing: 2 days**

This section describes the staining protocol to label mCherry, Ki67, CD45 and the nucleus of the cells of the lung sections.

7. Fixing and permeabilizing the lung sections.
  a. Place the slides on a slide-staining tray.
  b. Fix the slides in 4% formaldehyde in PBS for 10 minutes at 20°C–25°C.
    ***Note:*** The cells are already fixed from the inflation and incubation of the lungs with 4% formaldehyde. This step prevents the lung sections from detaching from the slides during the subsequent washing steps.
  c. Wash 3 times with PBS.
    ***Note:*** Perform each wash for 5 minutes at 20°C–25°C on a rocking platform with gentle sideways agitation. Use these settings for all subsequent washing steps in this section.
  d. Gently dry the region around the sections with a tissue.
  e. Encircle the dried region of the slide with a hydrophobic barrier pen (Vector Laboratories).
    ***Note:*** Make a single hydrophobic barrier on the slide, so that all 3 lung sections are within that area.
  f. Incubate the encircled region of the slides for 10 minutes at 20°C–25°C with 100 μL of the permeabilization solution.
    **CRITICAL:** This step is required for staining intracellular proteins, such as Ki67.
  g. Wash once with PBS containing 0.05% Tween 20.
8. Primary and secondary antibody stainings.
  a. Incubate the slides 12-16 hours at 4°C with 100 μL of the primary antibody mix.
    **CRITICAL:** Incubate the slide containing healthy, cancer cells-free lung sections (from here on referred to as the autofluorescence slide) with the unlabeled goat anti-mCherry antibody only.
    **CRITICAL:** Perform the incubations in a humidified chamber to prevent the sections from drying.
  b. Wash 3 times with PBS containing 0.05% Tween 20.
  c. Incubate the slides for 1 hour at 20°C–25°C with 70 μL of the secondary antibody mix.
    ***Note:*** Incubate the autofluorescence slide with the same secondary antibody mix. Perform this incubation in a humidified chamber to prevent the sections from drying.
  d. Wash 3 times with PBS containing 0.05% Tween 20.
  e. Incubate the slides for 5 minutes at 20°C–25°C with 100 μL of the nucleus staining solution.
    ***Note:*** Incubate the autofluorescence slide with 100 μL of water only.
  f. Wash with PBS containing 0.05% Tween 20.
  g. Place 3 drops of ProLong Diamond Antifade Mountant onto a cover slip.
  h. Hold the slide with the lung sections at an angle of 45°.
  i. Place the end of the slide onto the coverslip, allowing the first drop on the coverslip to touch the slide.
  j. Slowly lower the slide onto the coverslip.
  k. Firmly press the lateral edges of the slide onto the coverslip.
    **CRITICAL:** Do not press onto the tissue.
    ***Note:*** Remove any bubbles that may have formed by applying gentle pressure with a P1000 plastic tip.
  l. Store the slides at 4°C until the acquisition of immunofluorescence.

### Optimizing the exposure times of the fluorescence channels

**Timing: 1 h**

This section details the steps to adjust the exposure times of the fluorescence channels of the equipment to the intensity of the fluorescence in the samples at different magnifications.

9. Optimizing the exposure times of the fluorescence channels for whole slide scans.
  a. Open the software *Vectra 3.0.7.*
  b. Select “Edit protocol” → “Edit Filters and Bands…”.
  c. In “Whole Slide Scan Filters”, remove all the fluorescence channels except DAPI.
    ***Note:*** Only maintaining the DAPI channel at this stage significantly speeds up the whole slide scan process and contributes to an unbiased selection of the multispectral images at later steps. If you wish to have whole slide scans with more colors than DAPI, do not remove those channels.
  d. Load a positive control slide (i.e. lungs with 4T07-mCherry-positive metastases) onto the microscope.
  e. Select “Edit Exposures…”.
  f. Click on the exposure time of DAPI in the “Whole Scan Exposure (ms)” (Figure 4D).
  g. Locate a region with DAPI-positive cells.
  h. Select “Autofocus” (Figure 4D).
  i. Select “Autoexpose” (Figure 4D).
    **CRITICAL:** Only perform steps 9j-9o if other fluorescence channels were selected in step 9c.
  j. Locate a region on the slide with cells positive for a marker of interest.
  k. Switch to the DAPI channel.
  l. Perform “Autofocus” using the DAPI channel.
    **CRITICAL:** Do not select “Autoexpose” on DAPI again.
  m. Select the fluorescence channel of interest.
  n. Select “Autoexpose”.
    **CRITICAL:** Always repeat the “Autofocus” on the DAPI channel after moving the slide and before performing “Autoexpose” on the other channels.
  o. Repeat these steps for the other fluorescence channels.
10. Optimizing the exposure times of the fluorescence channels for multispectral images.
  a. Select “Edit Filters and Bands…”.
  b. In “Multispectral Regions Bands”, remove the channels for which no fluorophores are present in your staining panel.
  c. Select “Edit Exposures…”.
  d. Click on the exposure time of DAPI in the “MSI Regions Exposure (ms)” (Figure 4D).
  e. Locate a region with DAPI-positive cells.
  f. Select “Autofocus” (Figure 4D).
  g. Select “Autoexpose” (Figure 4D).
  h. Locate a region on the slide with cells positive for a marker of interest.
  i. Switch to the DAPI channel.
  j. Perform “Autofocus” using the DAPI channel.
    **CRITICAL:** Do not select “Autoexpose” on DAPI again.
  k. Select the fluorescence channel of interest.
  l. Select “Autoexpose”.
    **CRITICAL:** Always repeat the “Autofocus” on the DAPI channel after moving the slide and before performing “Autoexpose” on the other channels.
  m. Repeat these steps for the other fluorescence channels.
  n. Select “Save…” in the upper left corner to save the protocol with the new exposure times.
11. Taking a multispectral image of the autofluorescence slide.
  a. Remove the positive control slide from the microscope.
  b. Load the autofluorescence slide onto the microscope.
  c. Locate a region representative of normal lung tissue.
    **CRITICAL:** Do not perform any “Autofocus” or “Autoexpose”. Manually adjust the focus based on the autofluorescence of the sample.
  d. Select the column “MSI Regions Exposure (ms)”.
  e. Select “Snap” (Figure 4F).
    ***Note:*** This multispectral image will be used at later steps to remove the autofluorescence/background signal.

### Scanning the slides and acquiring multispectral images

**Timing: 2 days**

This section details the instructions to scan slides and select regions of interest from the scanned slides.

12. Acquiring whole slide scans.
  a. Encircle with a blue marker (on top of the coverslip) the lung sections to be screened.
    ***Note:*** Ensure the slide is clean before using the marker. If not, remove the dust/excess ProLong Diamond Antifade Mountant with a Cleantech wipe and ethanol.
  b. Load the slides to be analyzed into one tower.
  c. Place the tower with the slides in the first designated position on the Vectra machine (in the position of Cassette 1).
  d. Open *Excel.*
  e. Create an Excel file as indicated in Figure 5A (test task list.csv).

**Figure 5:**
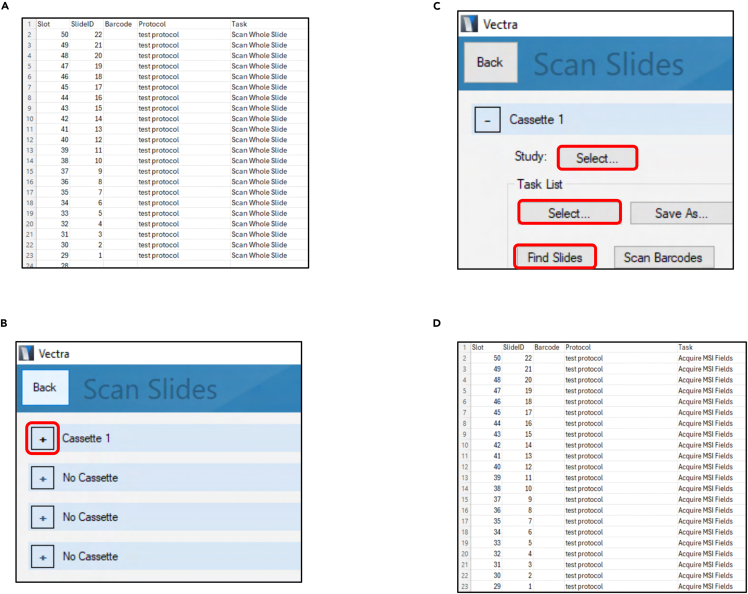
Procedure to perform whole slide scans and acquire multispectral images Sequential steps to automatically scan whole slides and automatically acquire multispectral images. Images adapted from screenshots of the software *Vectra 3.0.7* and *Excel.*
  f. Save the Excel file in the study where the protocol is saved.
  g. Select “Scan slides” in *Vectra 3.0.7.*
  h. Click on the “+” of Cassette 1 (Figure 5B).
  i. Select the study in which the protocol and the task list are saved (Figure 5C).
  j. Select the task list created on step 12e (Figure 5C).
  k. Select “Find Slides” (Figure 5C).
    ***Note:*** Wait for the robotic arm to find all the slides in the tower.
  l. Select “Process N slides”.
    ***Note:*** For problems with the whole slide scans, see Troubleshooting: Problem 4.
13. Selecting and acquiring multispectral images.
  a. Open the software *Phenochart 1.1.1.*
  b. Select “Login” in the upper right corner.
  c. Select “Load Slide” in the upper left corner.
  d. Open one whole slide scan.
  e. Toggle off all the channels except DAPI, on the bottom right (in case the slides were scanned with more fluorescence channels).
    **CRITICAL:** This step ensures the unbiased selection of random representative regions of the lungs.
  f. Select “Stamp”.
  g. Randomly stamp 15 regions per lung section (45 per slide).
    ***Note:*** The stamped regions are automatically saved.
  h. Repeat the process from step 13c with all whole slide scans.
  i. Close *Phenochart 1.1.1.*
  j. Update the “Task” column on the Excel task list as indicated in Figure 5D.
  k. Open the software *Vectra 3.0.7.*
  l. Repeat steps 12g-12i.
  m. Select the updated task list.
    ***Note:*** Confirm that the “Task” column in the Excel file reads “Acquire MSI Fields” before proceeding.
  n. Select “Find Slides”.
    ***Note:*** Wait for the robotic arm to find all the slides in the tower.
  o. Select “Process N Slides”.

### Training the algorithm and quantifying cancer cells

**Timing: 2 days**

This section provides the technical details to train the software to reproducibly identify and quantify 4T07-mCherry cells in the multispectral images.

14. Removing the autofluorescence from the multispectral images.
  a. Open the software *inForm 2.6.0.*
  b. Select “File” → “Open” → “Image” in the upper left corner.
  c. Randomly select a total of 10-15 multispectral images from different slides, including the one of the autofluorescence slide.
  d. Select “Sample Format: Fluorescence” (Figure 6A).

**Figure 6:**
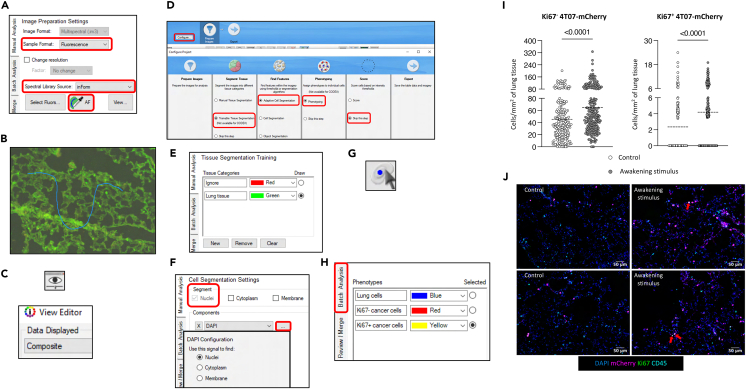
Quantification of cancer cells with an algorithm-based approach Overview of the workflow in *inForm 2.6.0* to train an algorithm to quantify disseminated cancer cells and the resulting plotted data and immunofluorescence images. The plotted data and the immunofluorescence images were adapted from Pereira et al.^1^ In the plots, each dot represents the density of cancer cells in a randomly chosen location in the lungs. Statistical analyses were performed with a Mann-Whitney test. The mean is represented by a dashed line. Red arrows indicate Ki67^+^ 4T07-mCherry cells in the immunofluorescence images. Images adapted from screenshots of the software *Vectra 3.0.7* and *Graph Pad Prism*.
  e. Select “Spectral Library Source: inForm” (Figure 6A).
  f. Tick the fluorophores used in the staining panel.
  g. Select the autofluorescence multispectral image.
  h. Select the “AF” tool, as indicated in Figure 6A.
  i. Draw a line onto a representative section of the lungs on the autofluorescence multispectral image (Figure 6B).
    ***Note:*** This step establishes a baseline for the autofluorescence in all multispectral images.
  j. Select “Prepare all” in the bottom left corner to remove the autofluorescence in all the loaded multispectral images.
15. Segmenting the tissue and the cells.
  a. Select a random multispectral image (not the autofluorescence image).
  b. Click on the “View Editor” icon and select “Data Displayed: Composite” (Figure 6C).
    ***Note:*** Adjust the colors/intensities of the fluorescence channels in the image at your preference.
  c. Select “Configure” and tick the options shown in Figure 6D.
  d. Select “Advance” in the bottom left corner.
  e. Select “New” and add “Tissue Categories”.
  f. Mark the regions to be considered as “Lung tissue” and “Ignore” (Figure 6E).
    ***Note:*** The regions defined as “Ignore” consist of empty spaces and the highly autofluorescent cells lining vessels and bronchioles).
    **CRITICAL:** Mark both regions on different multispectral images to increase the accuracy of the training algorithm.
  g. Select “Train Tissue Segmenter”.
  h. Wait until the training accuracy stabilizes between 95%–100%.
    ***Note:*** The time and accuracy of the training depend on the chosen “Pattern Scale” and “Segmentation Resolution”.
  i. Select “Segment All” and “Advance”.
  j. Select “Segment: Nuclei” → “Components: DAPI” → “…” (Figure 6F).
  k. Select “Nuclei” and adjust the “Typical Intensity (Relative)” based on the DAPI staining.
  l. Adjust the “Splitting Sensitivity” and “Minimum Nuclear Size” according to the characteristics of the cells.
  m. Select “Segment All” and “Advance”.
16. Identifying cell phenotypes.
  a. Select “Add” and insert the phenotypes to be identified in the multispectral images.
    ***Note:*** Choose colors that do not conflict with those selected in step 15b.
  b. Select the icon to manually phenotype cells (Figure 6G).
  c. Click on a cell and select its phenotype, based on its signature of fluorophores.
    **CRITICAL:** Identify a minimum of 5 cells per phenotype. Identifying more cells per phenotype improves the accuracy of the “Phenotype Classifier”.
  d. Select “Train Classifier”.
  e. Select “Phenotype All”.
  f. Check the accuracy of the phenotype classifier on all the loaded multispectral images.
    **CRITICAL:** If cells are not correctly phenotyped by the algorithm, repeat steps 16b and 16c.
    ***Note:*** Manually phenotype the cells that were wrongly phenotyped by the algorithm to improve the new algorithm’s accuracy.
  g. Retrain the classifier and select “Phenotype All”.
    **CRITICAL:** Repeat these steps until all cells are correctly phenotyped.
17. Quantifying cell phenotypes.
  a. On “View Editor”, select “Data Displayed: Tissue Segmentation Data” and tick the desired “Table Contents”.
  b. On “View Editor”, select “Data Displayed: Cell Segmentation Data” and tick the desired “Table Contents”.
  c. On the left side, select “Batch Analysis” (Figure 6H).
  d. Select “Export Directory: Browse…” and create a new folder to store the batch analysis.
  e. Tick the desired “Image export options”.
  f. Select “Use view settings” to export the table fields selected on steps 17a and 17b.
  g. Select “Add Slides…” and choose all whole slide scans.
    ***Note:*** All 45 multispectral images from each slide are automatically retrieved.
  h. Select “Run”.
  i. Select “Review Merge” on the left side, after the batch analysis is complete.
  j. Confirm that the phenotyping was done accurately.
    **CRITICAL:** If not, go back to step 16b and proceed thereafter.
  k. Select “Merge” and save the files in the same folder.
  l. Open the Merged text file on *Excel.*
  m. Plot the density of Ki67-negative and Ki67-positive 4T07-mCherry cancer cells in the lungs (Figure 6I).
  n. Add representative immunofluorescence images of the plotted data (Figure 6J) (red arrows indicate Ki67-positive 4T07-mCherry cells).

## Expected outcomes

### Preparation of 4T07-mCherry breast cancer cells for injection

The conditioned medium of *in vitro*-cultured 4T07-mCherry cells should be pinkish, but not yellowish. Cells should be passaged once after thawing before injection, and collected during the phase of exponential growth. At the end of this step, you should have a cell suspension containing 2.0x10^6^ viable 4T07-mCherry cells/mL of PBS.

### Injection of 4T07-mCherry cells into the mammary fat pad

The injection of 4T07-mCherry cells into the 4^th^ mammary fat pad leads to the formation of a bulge in the fatty tissue, which is no longer visible once the mouse awakens and starts walking. No bleeding is expected during and after the injection. Mice often lick the site of injection on the first day. At the end of this step, 4T07-mCherry cancer cells have been injected into the 4^th^ mammary fat pad and mice resumed walking normally.

### Resection of the primary tumor from the mammary fat pad

The primary breast tumor is resected 21 days after injection of breast cancer cells into the mammary fat pad. No bleeding is expected when cutting the skin around the tumor. Bleeding is expected when cutting large blood vessels connected to the tumor, even when using hemostatic forceps to clamp the vessels before cutting. Signs of pain after surgery are expected to subside after a couple of days. Once the wound is closed, mice begin removing the staples and stitches on their own. This does not require re-stapling nor re-stitching the wound (considering the wound has already healed and closed). The fur will regrow on the shaved area. At the end of this step, the primary tumor has been resected.

### Collection of lungs

From mice that don’t undergo resection of the primary tumor, the lungs are collected on day 21 after the injection of 4T07-mCherry cells in the mammary fat pad. From mice that underwent resection of the primary tumor (see above), the lungs can be collected at any time after resection depending on the research question. We usually waited at least one week after resection to exclude analysis of recently disseminated (and therefore, not necessarily dormant) cancer cells. No metastases are expected to be present in other organs, both before and after the resection of the 4T07-mCherry tumor. At the end of this step, the collected lungs have been fixed and incubated in solutions with different concentrations of sucrose.

### Freezing of lungs

The lungs should be completely embedded in the O.C.T. Compound and therefore not visible after freezing. At the end of this step, the lungs and the block are frozen and at the same temperature.

### Processing and staining the lungs for immunofluorescence

The processing and staining procedures will remove the O.C.T. Compound from the slides, leaving only the sections and the hydrophobic barrier on the slides. At the end of this step, the lung sections have been stained with the primary and secondary antibodies and the immunofluorescence is ready to be registered.

### Preparing the protocol for the detection of immunofluorescence

The fluorescence references are always acquired before preparing/importing a protocol, so variations in the light beam and fluorescence channels on different days are not expected to affect the data. At the end of this step, a protocol with optimized exposure times has been elaborated and a snapshot of the autofluorescence has been taken.

### Scanning slides and acquiring multispectral images

A total of N whole slide scans and 45N multispectral images are expected after running the generated task lists on *Vectra 3.0.7.* At the end of this step, the multispectral images are ready to be analyzed with *inForm 2.6.0.*

### Quantification of cancer cells with an algorithm-based approach

The text file generated after selecting “Merge” will contain the quantification of all identified phenotypes in the selected “Tissue Categories”. At the end of this step, the data selected in *inForm 2.6.0* is available in a text file that can be opened with *Excel.*

## Limitations

This manuscript describes the sequential steps required to study the three steps of metastatic dormancy: induction, maintenance, and awakening. The described resection of the primary tumor is a delicate process that requires practice and care and is a source of pain and discomfort to mice. First performing surgery on euthanized mice provides a means to practice the procedure and thus avoid making mistakes that add to the suffering associated with the surgery. The resection of the primary tumor to study the maintenance of and awakening from metastatic dormancy can only be performed when using breast cancer cells of which DCCs are dormant. Breast cancer cell lines that disseminate to and proliferate in other organs make the resection of the primary tumor insufficient to eliminate the source of cancer cells. The phenotype of the cells of interest is identified based on the presence/absence of fluorophores. This protocol enables the use of 4 different fluorophores (plus DAPI), which limits the phenotypes that can be identified. Using other, more sophisticated lung processing and staining protocols permits more fluorophores to be used and detected with the same software, increasing the number of phenotypes that can be identified.

## Troubleshooting

### Problem 1

The mammary fat pad is difficult to see in thin mice.

### Potential solution

Place your thumb over the nipple of the 4^th^ mammary fat pad and the index on the opposite side of the skin. Insert the needle next to the nipple, close to your thumb (as demonstrated in Methods video S1) and slowly inject the injection mix. This alternative site of injection does not require visualization of the mammary fat pad and delivers cancer cells into the ventral region of the 4^th^ mammary fat pad.

Methods video S1. Demonstration of the injection of 4T07-mCherry cancer cells into the right 4th mammary fat pad

### Problem 2

The incision is too deep and the peritoneum is cut.

### Potential solution

Suture the peritoneum (never staple) and remove all ends of the stitches after making a knot to prevent discomfort to the mouse. Proceed with the surgery.

### Problem 3

One lung is not inflating/both lungs do not inflate.

### Potential solution

• One lung is not inflating: The plastic cannula is too deep. Pull it up a few millimeters and inflate again.
• One lung continues not to inflate/both lungs are not inflating: The lungs were pierced during collection. Inflation with 4% formaldehyde is no longer possible, but the lungs will still be fixed by proceeding to the next steps.

### Problem 4

One slide (or more) is not scanned after running the program.

### Potential solution

• The blue line delineating the sections is defective. Erase this line with a tissue damped in 100% ethanol (do not move the coverslip while doing so) and draw a new line without lifting the marker. Ensure the line forms a closed area, don’t leave gaps. Re-scan the slide(s).
• The upper side of the coverslip/bottom side of the slide are dirty with dust and/or O.C.T. Compound. Gently clean both surfaces with a tissue damped in 100% ethanol. Re-draw the blue line if needed. Re-scan the slide(s).

## Resource availability

### Lead contact

Further information and requests for resources and reagents should be directed to and will be fulfilled by the lead contact, Maries van den Broek (maries.vandenbroek@uzh.ch).

### Technical contact

Further information and requests about the experimental procedures and analysis of data should be directed to and will be fulfilled by the technical contact, Paulo Pereira (paulobp1998@gmail.com).

### Materials availability

This protocol does not generate new unique reagents.

### Data and code availability

This protocol does not generate new unique reagents.

## Acknowledgments

This work was supported by the University Research Priority Program ‘‘Translational Cancer Research’’ (University of Zurich; M.v.d.B.), SKINTEGRITY.CH (University of Zurich; M.v.d.B.), the Hartmann-Müller Foundation (P.P.), the Swiss National Science Foundation (310030_208145; M.v.d.B.), and the Monique-Dornonville-de-la-Cour-Foundation (M.v.d.B.). We thank Dr. Anna Laura Calvanese (University of Zurich) and Dr. Karina Silina (ETH Zurich) for initial help with analyzing immunofluorescence. The authors thank the personnel of the Laboratory Animal Services Center (LASC, University of Zurich) and the Zurich Integrative Rodent Physiology (ZIRP, University of Zurich) for expert animal care and the Center for Microscopy and Image Analysis (ZMB, University of Zurich) for excellent support.

## Author contributions

P.P., V.C., and M.v.d.B. conceived the experiments. P.P. and M.v.d.B. wrote the manuscript. M.v.d.B. secured funding. All authors reviewed and approved the final manuscript.

## Declaration of interests

The authors declare no competing interests.

## Declaration of generative AI and AI-assisted technologies in the writing process

Images of mice, surgery tools, material, algorithm, and organs were obtained by Generative AI. The images of the described procedures were manually elaborated using the generated images. All images of the software were obtained by screenshots of the respective program. The image of the microscope was obtained by photograph.
